# Genomic analysis of four strains of *Corynebacterium pseudotuberculosis* bv. Equi isolated from horses showing distinct signs of infection

**DOI:** 10.1186/s40793-017-0234-6

**Published:** 2017-01-31

**Authors:** Rafael A. Baraúna, Rommel T. J. Ramos, Adonney A. O. Veras, Pablo H. C. G. de Sá, Luís C. Guimarães, Diego A. das Graças, Adriana R. Carneiro, Judy M. Edman, Sharon J. Spier, Vasco Azevedo, Artur Silva

**Affiliations:** 1 Federal University of Pará, Institute of Biological Sciences, Center of Genomics and Systems Biology, Belém, Brazil; 2 Department of Medicine and Epidemiology, School of Veterinary Medicine, University of California, Davis, CA USA; 3 Federal University of Minas Gerais, Institute of Biological Sciences, Laboratory of Cellular and Molecular Genetics, Belo Horizonte, Brazil

**Keywords:** *C. pseudotuberculosis*, Biovar equi, Ulcerative lymphangitis, Horse, Genomic

## Abstract

The genomes of four strains (MB11, MB14, MB30, and MB66) of the species *Corynebacterium pseudotuberculosis* biovar equi were sequenced on the Ion Torrent PGM platform, completely assembled, and their gene content and structure were analyzed. The strains were isolated from horses with distinct signs of infection, including ulcerative lymphangitis, external abscesses on the chest, or internal abscesses on the liver, kidneys, and lungs. The average size of the genomes was 2.3 Mbp, with 2169 (Strain MB11) to 2235 (Strain MB14) predicted coding sequences (CDSs). An optical map of the MB11 strain generated using the KpnI restriction enzyme showed that the approach used to assemble the genome was satisfactory, producing good alignment between the sequence observed in vitro and that obtained *in silico*. In the resulting Neighbor-Joining dendrogram, the *C. pseudotuberculosis* strains sequenced in this study were clustered into a single clade supported by a high bootstrap value. The structural analysis showed that the genomes of the MB11 and MB14 strains were very similar, while the MB30 and MB66 strains had several inverted regions. The observed genomic characteristics were similar to those described for other strains of the same species, despite the number of inversions found. These genomes will serve as a basis for determining the relationship between the genotype of the pathogen and the type of infection that it causes.

## Introduction

As of February 2016, thirty-three genomes of the species *Corynebacterium pseudotuberculosis* had been deposited into the National Center for Biotechnology Information database. This species is an animal pathogen that infects goats and sheep, causing caseous lymphadenitis, as well as horses, which can show distinct signs and symptoms. *C. pseudotuberculosis* can be classified into two biovars based on its ability to reduce nitrate to nitrite [1]. Non-reducing, i.e., nitrate-negative, strains are grouped into the ovis biovar and are responsible for CL. The reducing, i.e., nitrate-positive, strains are grouped into the equi biovar and mainly infect horses.

Recent increases in the number of infections in horses have led to *C. pseudotuberculosis* bv. equi being classified as a re-emerging pathogen. In Texas, USA, the number of cases increased 10-fold between 2005 and 2011, with a cumulative increase in annual incidence from 9.3 to 99.5 infections per 100,000 horses over the same period [2]. Kilcoyne et al. [3] analyzed the number of cultures positive for *C. pseudotuberculosis* in samples isolated from infected horses in 23 states in the USA. The proportion of positive cultures was higher for the most recent years, 2011 and 2012 (54% of the total number of samples), than for the period spanning 2003 to 2010 (46% of the total number of samples). These current data show the growing numbers of infections caused by this bacterium and emphasize the need for new studies on the genotypic characteristics of the biovar.

*C. pseudotuberculosis* bv. equi infection is commonly known as “pigeon fever” because it leads to the formation of external abscesses on the chest of the animal, making it expand, similar to a pigeon breast. Despite its common name, the bacteria can also cause other types of infections with distinct signs and symptoms, such as the formation of internal abscesses or ulcerative lymphangitis, which is characterized by the infection of limbs and compromises the lymphatic system [4]. It is currently believed that the major vectors of the disease are domestic flies of the species *Haematobia irritans*, *Stomoxys calcitrans* *,* and *Musca domestica* [5].

The pathogenesis of *C. pseudotuberculosis* is intrinsically linked to its genetic content. Several virulence factors have previously been described in the literature that strongly influence the ability of the bacteria to interact with the host, causing infection. Phospholipase D, the iron uptake system, and pili proteins are examples of these factors [6]. Characterization of these and novel virulence factors depends on the sequencing of new genomes from the biovar, as the vast majority of the genomes in databases belong to the ovis biovar. Therefore, to generate data that allows for a more robust genotypic analysis of the equi biovar, four genomes from strains isolated from horses with distinct signs of infection by *C. pseudotuberculosis* were sequenced using the next-generation Ion Torrent PGM platform.

## Organism information

### Classification and features

**Fig. 1:**
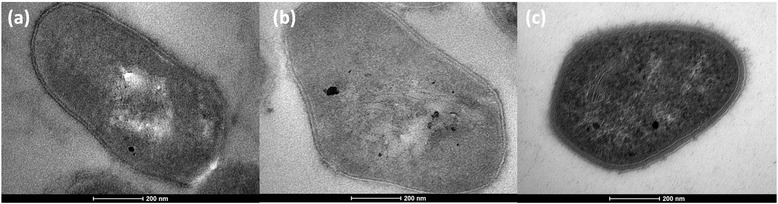
Transmission Electron Micrograph of three strains sequenced in this study. The electron micrographs of **a** MB11, **b** MB30 and **c** MB66, demonstrate the pleomorphic morphology of the species

*C. pseudotuberculosis* bv. equi is a facultative intracellular, beta-hemolytic, pleomorphic (Fig. 1), non-sporulating, unencapsulated, non-mobile, facultative anaerobic, Gram-positive pathogen. [6]. The main characteristics of the species are shown in Table 1. *C. pseudotuberculosis* is taxonomically classified in the phylum *Actinobacteria*, class *Actinobacteria*, order *Corynebacteriales*, family *Corynebacteriaceae*, and genus *Corynebacteria*. The strains included in this study were isolated from horses in the state of California, USA. The animals had distinct signs and symptoms of infection. Strain MB11 was isolated from a 6-month-old American Paint horse with ulcerative lymphangitis. Strain MB14 was isolated from an Arab/Saddle horse with abscess formation in internal organs (liver and kidney). The animal also presented hepatic lipidosis and myocardial fibroses. Strain MB30 was isolated from the pectoral abscess of a 2-year-old Quarter horse. Finally, strain MB66 was isolated from a 20-year-old Polish Arab mare with metastatic melanoma and multiple external and internal abscesses. These distinct signs, such as pectoral abscesses (“pigeon fever”), abscesses on the internal organs, or abscesses on the limbs (ulcerative lymphangitis), suggest that the equi biovar can interact in several ways with the host animal to cause infection. All strains were isolated over the period of October-1996 up to June-2002.

**Table 1:** Classification and general features of the species strain designation^T^ [cite MIGS reference]

MIGS ID	Property	Term	Evidence code^a^
	Classification	Domain: *Bacteria*	TAS [22]
		Phylum: *Actinobacteria*	TAS [23]
		Class: *Actinobacteria*	TAS [24]
		Order: *Corynebacteriales*	TAS [25, 26]
		Family: *Corynebacteriaceae*	TAS [27, 28]
		Genus: *Corynebacterium*	TAS [28, 29]
		Species: *C. pseudotuberculosis*	TAS [28, 30]
		strain: MB11, MB14, MB30 and MB66	IDA
	Gram stain	Positive	TAS [31]
	Cell shape	Pleomorphic	TAS [31]
	Motility	Non-motile	TAS [31]
	Sporulation	Non-sporulated	TAS [31]
	Temperature range	Mesophilic	TAS [32]
	Optimum temperature	37 °C	TAS [32]
	pH range; optimum	7.0–7.2	TAS [32]
	Carbon source	Glucose, fructose, maltose, mannose, and sucrose	TAS [6]
MIGS-6	Habitat	Soil and animal pathogens	TAS [4, 33]
MIGS-6.3	Salinity	Up to 2 M NaCl	TAS [32]
MIGS-22	Oxygen requirement	Facultative anaerobe	TAS [6]
MIGS-15	Biotic relationship	Intracellular facultative pathogen	TAS [6]
MIGS-14	Pathogenicity	*Equus caballus*	TAS [4]
MIGS-4	Geographic location	California, USA	IDA
MIGS-5	Sample collection	MB11: Oct-96 MB14: Dec-96 MB30: Nov-00 MB66: Jun-02	IDA
MIGS-4.1	Latitude	MB11 - 38°21′23″ MB14 - 37°00′20″ MB30 - 39°39′32″ MB66 - 38°32′41″	IDA
MIGS-4.2	Longitude	MB11 - 121°59′15″ MB14 - 121°34′05″ MB30 - 121°37′52″ MB66 - 121°44′25″	IDA
MIGS-4.4	Altitude	MB11 - 180 ft MB14 - 196 ft MB30 - 351 ft MB66 - 55 ft	IDA

^a^Evidence codes - *IDA* Inferred from Direct Assay, *TAS* Traceable Author Statement (i.e., a direct report exists in the literature), *NAS* Non-traceable Author Statement (i.e., not directly observed for the living, isolated sample, but based on a generally accepted property of the species or anecdotal evidence). These evidence codes are from the Gene Ontology project [cite this reference]

**Fig. 2:**
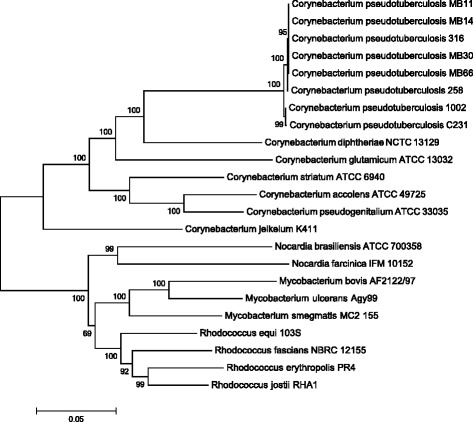
Dendrogram of the representative genomes in the CMNR group. The analysis was performed using MEGA 5.10. Only bootstraps greater than 50% are shown in the branches of the dendrogram. The accession numbers for the sequences used in the analysis are: *C. pseudotuberculosis* MB11 (CP013260), *C. pseudotuberculosis* MB14 (CP013261), *C. pseudotuberculosis* MB30 (CP013262), *C. pseudotuberculosis* MB66 (CP013263), *C. pseudotuberculosis* 316 (CP003077), *C. pseudotuberculosis* 258 (CP003540), *C. pseudotuberculosis* 1002 (CP001809), *C. pseudotuberculosis* C231 (CP001829), *C. diphtheriae* NCTC 13129 (BX248353), *C. glutamicum* ATCC 13032 (BA000036), *C. striatum* ATCC 6940 (GCA_000159135), *C. accolens* ATCC 49725 (GCA_000159115), *C. pseudogenitalium* ATCC 33035 (NZ_ABYQ00000000), *C. jeikeium* K411 (NC_007164), *N. brasiliensis* ATCC 700358 (CP003876), *N. farcinica* IFM 10152 (NC_006361), *M. bovis* AF2122/97 (BX248333), *M. ulcerans* Agy99 (CP000325), *M. smegmatis* MC2 155 (CP000480), *R. equi* 103S (FN563149), *R. fascians* NBRC 12155 (GCA_001894785), *R. erythropolis* PR4 (NC_012490), *R. jostii* RHA1 (NC_008268)

A dendrogram was calculated with the Neighbor-joining statistical method using a bootstrap analysis with 1000 replicates. The *rpoB* gene, which codes for the beta subunit of the RNA polymerase enzyme, was used as a marker when constructing the dendrogram. The analysis was performed using the NCBI reference sequence for the species, retrieving from the database at least one representative from each genus in the *Corynebacterium*, *Mycobacterium*, *Nocardia* *,* and *Rhodococcus* group (Fig. 2). This group is composed of species that share cellular characteristics, such as a cell wall composed of peptidoglycan, arabinogalactan, and mycolic acids, as well as a genome with a high GC content [6]. The first phylogenetic studies on the CMNR group used the 16S rRNA gene as a marker. These studies demonstrated that the genera in the family *Corynebacteriaceae* form a monophyletic clade composed of four groups, in which *C. pseudotuberculosis* is phylogenetically closest to the species *C. ulcerans* and *C. diphtheriae* [7]. Recently, Khamis et al. [8] proposed that the gene *rpoB* could be used as a marker to identify clinical isolates of the genus *Corynebacterium*. The positive results for identification using the *rpoB* gene were greater than those of the 16S rRNA gene, indicating that *rpoB* is useful for taxonomic classification the family *Corynebacteriaceae* [8]. The dendrogram in Fig. 2 shows the phylogenetic proximity between the sequenced biovars of the species *C. pseudotuberculosis*. In addition, it corroborates the analyses performed with the 16S rRNA gene, which designated *C. diphtheriae* as the species most closely related to *C. pseudotuberculosis*. The results show that each genus in the CMNR group is divided into clades supported by high bootstrap values.

## Genome sequencing information

### Genome project history

The four *C. pseudotuberculosis* genomes in this short report are part of a collaboration between the University of California, Davis, USA, and the Federal Universities of Minas Gerais and Pará, Brazil. The project seeks to determine the genomic characteristics of 12 strains of the equi biovar isolated from horses in California showing distinct signs and symptoms of infection. Isolation was performed over several years from different horse breeds (Table 2). One of the major aims of the project is to determine if a relationship exists between the genetic content of the strains and the type of infection that it causes (i.e., ulcerative lymphangitis, external abscesses, or internal abscesses). In parallel, the project seeks to increase the amount of genomic data for the species *C. pseudotuberculosis* in databases, which will form the basis for broader functional studies. The genomes obtained in this study have been deposited into the NCBI database under accession number CP013260, CP013261, CP013262, CP013263. The project information is also presented in Table 2.

**Table 2:** Project information

MIGS ID	Property	Term
MIGS 31	Finishing quality	Completed
MIGS-28	Libraries used	Fragments library
MIGS 29	Sequencing platforms	Ion Torrent PGM
MIGS 31.2	Fold coverage	842x (MB11); 867x (MB14); 309x (MB30); 658x (MB66).
MIGS 30	Assemblers	MIRA4, Lasergene (DNASTAR), GapBlaster.
MIGS 32	Gene calling method	Pannotator (FgenesB; Glimmer; tRNAscan; RNAmer)
	Locus Tag	ATN02_ (MB11); ATN03_ (MB14); ATN04_ (MB30); ATN05_ (MB66)
	GenBank ID	CP013260 (MB11); CP013261 (MB14); CP013262 (MB30); CP013263 (MB66).
	GenBank Date of Release	2016-03-01
	GOLD ID	Gp0131493 (MB11); Gp0131495 (MB14); Gp0131496 (MB30); Gp0131497 (MB66).
	BIOPROJECT	PRJNA256958
MIGS 13	Source Material Identifier	Isolated directly from the infected animal
	Project relevance	Animal pathogen

### Growth conditions and genomic DNA preparation

After isolation, the bacteria were maintained in 25% glycerol at −80 °C, and the medium was refreshed routinely. To extract genomic DNA, the bacteria were first cultured in liquid brain heart infusion (BHI) medium at 37 °C with shaking. DNA was extracted during the log-phase of cell growth according to the protocol described by Pacheco et al. [9] for clinical isolates. The extracted DNA was subjected to electrophoresis on a 1% agarose gel to determine the quality of the material.

### Genome sequencing and assembly

**Fig. 3:**
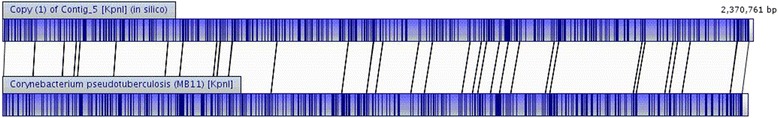
Optical map of *Corynebacterium pseudotuberculosis* MB11. The figure shows the alignment of the KpnI sites observed in the optical map (*bottom scale bar*) with those predicted by the *in silico* assembly (*top scale bar*). Vertical lines connect identical restriction sites observed in the optical map and those predicted by the assembly, demonstrating that the genome was assembled in the correct order

Genomic DNA was sequenced on the Ion Torrent PGM (Thermo Scientific) platform using the 318 chip v2 in accordance with the manufacturer’s instructions. The quality of the reads was analyzed using FastQC software [10]. The reads were then trimmed and filtered to remove those with a phred-scaled quality score less than 20. Next, the reads were assembled using Mira 4 software [11]. Redundancy within the assembled contigs was eliminated using the SeqMan Pro tool in the Lasergene software package (DNASTAR). The few remaining gaps after redundancy removal were manually closed using local BLAST or a program developed by our research group called GapBlaster [12], which uses a reference genome to assemble similar sequences to close the gap using the sequencing reads. For this analysis, we used *C. pseudotuberculosis* biovar equi strain 316 as a reference. An optical map using KpnI restriction sites was generated to evaluate the quality of the genome assembly for the MB11 strain (Fig. 3). The optical map was analyzed using MapSolver v.3.2.0 (OpGen). Figure 3 shows that the *in silico* assembly for strain MB11 was very satisfactory; the positions of the restriction sites were corroborated by the optical map analysis.

### Genome annotation

An automatic annotation was first conducted using the online software Pannotator [13], which provided the .fasta files for the assembled genomes and a reference .embl file for *C. pseudotuberculosis* 316. The results were then manually curated to meet the gene annotation standards set by UniProt [14] using Artemis software [15] to visualize the coding sequences. Next, pseudogenes were also manually curated to resolve mismatches using CLC Genomics Workbench 5 (CLC Bio) and Artemis. Predicted genes for the four genomes were classified by the clusters of orthologous groups functional category, as shown in Table 3.

**Table 3:** Number of genes associated with general COG functional categories

Code	MB11		MB14		MB30		MB66		Description
	Value	%age	Value	%age	Value	%age	Value	%age	
J	127	5.83	148	6.62	123	5.64	122	5.54	Translation, ribosomal structure, and biogenesis
A	1	0.05	1	0.04	1	0.05	1	0.05	RNA processing and modification
K	55	2.52	90	4.03	55	2.52	54	2.45	Transcription
L	63	2.89	96	4.29	67	3.07	66	3.00	Replication, recombination, and repair
B	0	0	0	0	0	0	0	0	Chromatin structure and dynamics
D	16	0.73	25	1.12	16	0.73	16	0.73	Cell cycle control, cell division, and chromosome partitioning
V	13	0.60	23	1.03	13	0.60	13	0.59	Defense mechanisms
T	17	0.78	55	2.46	17	0.78	16	0.73	Signal transduction mechanisms
M	55	2.52	82	3.67	55	2.52	54	2.45	Cell wall/membrane biogenesis
N	1	0.05	14	0.63	1	0.05	1	0.05	Cell motility
U	17	0.78	21	0.94	17	0.78	17	0.77	Intracellular trafficking and secretion
O	53	2.43	79	3.53	55	2.52	53	2.41	Posttranslational modification, protein turnover, and chaperones
C	73	3.35	121	5.41	74	3.40	73	3.32	Energy production and conversion
G	73	3.35	100	4.47	74	3.40	72	3.27	Carbohydrate transport and metabolism
E	122	5.60	180	8.05	122	5.60	122	5.54	Amino acid transport and metabolism
F	58	2.66	74	3.31	57	2.62	57	2.59	Nucleotide transport and metabolism
H	83	3.81	113	5.05	83	3.81	83	3.77	Coenzyme transport and metabolism
I	36	1.65	51	2.28	36	1.65	35	1.59	Lipid transport and metabolism
P	68	3.12	118	5.28	67	3.07	67	3.04	Inorganic ion transport and metabolism
Q	13	0.60	28	1.25	13	0.60	12	0.55	Secondary metabolite biosynthesis, transport, and catabolism
R	113	5.19	275	12.30	111	5.09	110	5.00	General function prediction only
S	112	5.14	153	6.84	112	5.14	113	5.13	Function unknown
-	1010	46.35	389	17.40	1056	46.35	1044	47.43	Not in COGs

The total is based on the total number of protein coding genes in the genome

## Genome properties

All of the genomes were completely closed, resulting in a size of 2,363,423 bp for strain MB11, 2,370,761 bp for MB14, 2,364,377 for MB30, and 2,372,202 bp for MB66. The approximately 2.3 Mbp size is similar to other previously studied and published equi strains [16–18]. Four ribosomal RNA clusters were observed in all of the genomes. The strains had an average GC content of 52% and a total of 51 tRNAs predicted by tRNAscan-SE for each strain [19]. MB11 had a total of 2179 CDSs and 37 pseudogenes after manual curation. MB14 had 2235 CDSs and 20 pseudogenes, while MB30 had 2225 CDSs and six pseudogenes, and finally, MB66 had 2201 CDSs and 54 pseudogenes. A more detailed description of the genomic statistics is presented in Table 4.

**Table 4:** Genome statistics

Attribute	MB11		MB14		MB30		MB66	
	Value	% of Total	Value	% of Total	Value	% of Total	Value	% of Total
Genome size (bp)	2,363,423	100.0	2,370,761	100.0	2,364,377	100.0	2,372,202	100.0
DNA coding (bp)	2,021,172	85.52	2,052,709	86.58	2,066,802	87.41	2,006,473	84.58
DNA G + C (bp)	1,067,329	52.09	1,235,085	52.1	1,231,731	52.09	1,235,856	52.1
DNA scaffolds	1	100.0	1	100.0	1	100.0	1	100.0
Total genes	2,260	100.0	2,317	100.0	2,237	100.0	2,334	100.0
Protein coding genes	2,179	96.41	2,235	96.46	2,225	99.46	2,201	94.30
RNA genes	63	2.79	63	2.78	63	2.82	63	2.70
Pseudo genes	37	1.64	20	0.86	6	0.27	54	2.31
Genes in internal clusters	775	34.29	785	33.88	779	34.82	774	33.16
Genes with function prediction	1,526	67.52	1,576	68.02	1,577	70.50	1,550	66.41
Genes assigned to COGs	1,169	51.72	1,847	79.71	1,169	52.26	1,157	49.57
Genes with Pfam domains	1,722	76.19	1,819	80.49	1,823	78.68	1,797	76.99
Genes with signal peptides	88	3.89	92	3.97	93	4.16	86	3.68
Genes with transmembrane helices	589	26.06	607	26.20	604	27.00	583	24.98
CRISPR repeats	3	0.01	3	0.01	3	0.01	2	0.01

**Fig. 4:**
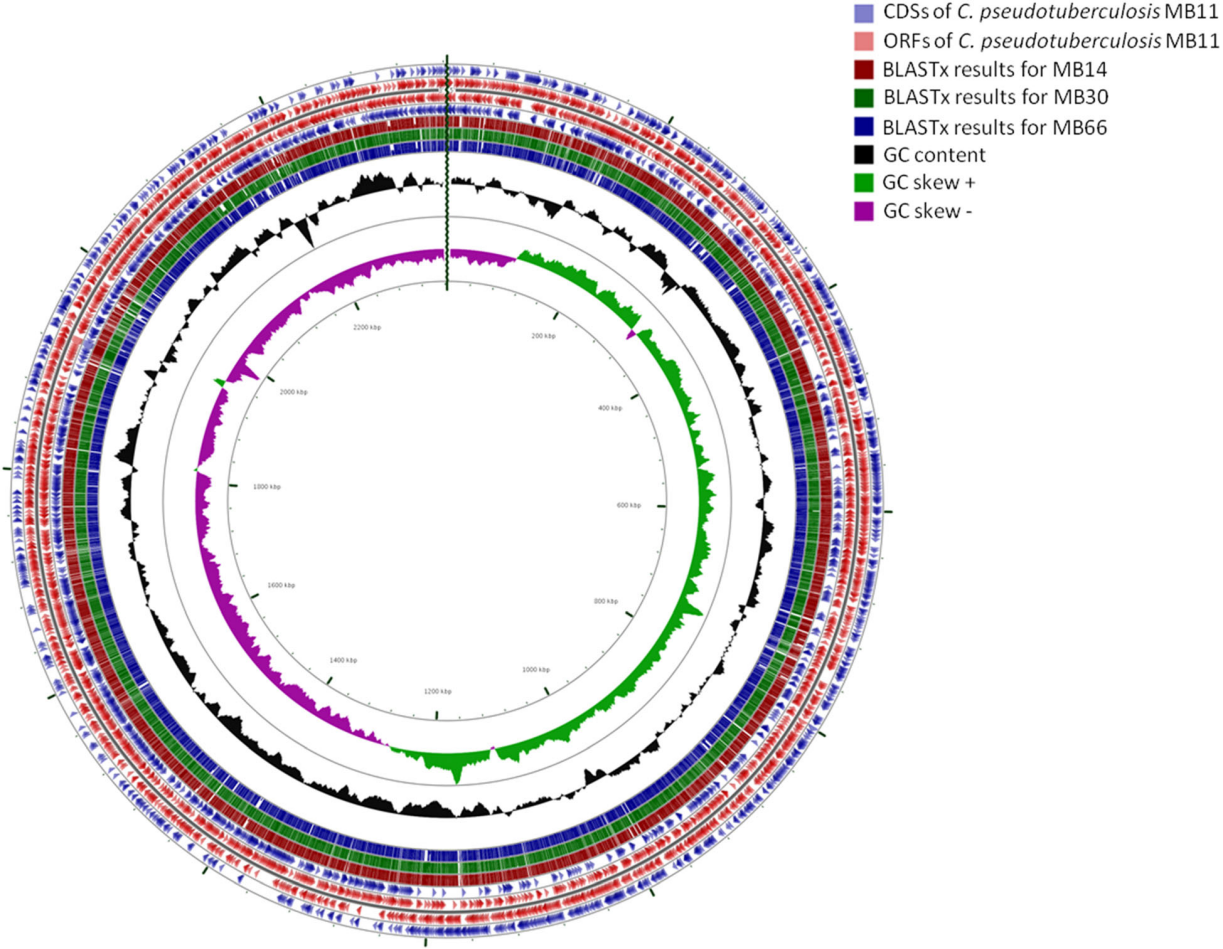
Circular map of the genome for the sequenced *Corynebacterium pseudotuberculosis* strains. The outermost ring in *blue* shows the features extracted from the MB11 genome using a .gbk file. The next ring shows the CDSs predicted on the forward strand of MB11 in *red*, followed by the CDSs on the reverse strand with their features in *blue*. The other three rings in *red*, *green*, and *blue* show proteins predicted by blastx for the MB14, MB30, and MB66 genomes, respectively, compared to the MB11 genome. The two innermost rings show the GC content and GC skew, followed by the size of the genome in base pairs

**Fig. 5:**
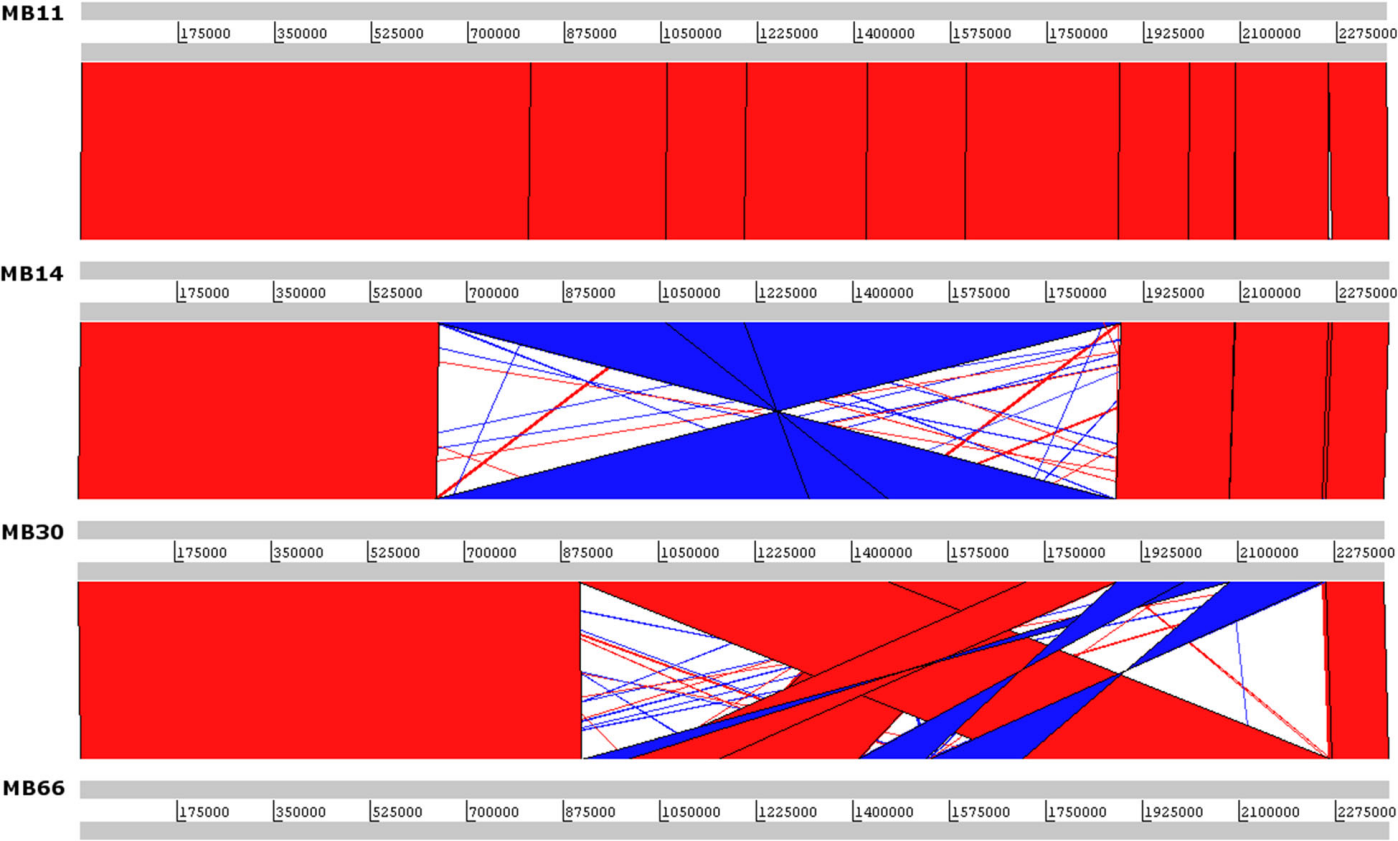
Comparison of *C. pseudotuberculosis* genome structures using blastn. The names of the strains are indicated at the side of the *gray bars* showing the size of each genome. *Red bars* show conserved regions between two genomes using an e-value of 1-e05, while *blue bars* show inverted regions

A circular map was generated using the CGView web tool [20] that shows the relationship of the predicted proteins in the MB14, MB30, and MB66 genomes compared to strain MB11, in which the *in silico* assembly was corroborated by the optical map (Fig. 4). All of the genomes had similar sizes and a similar number of CDSs, with few differences between the coding regions of the genomes. Structural analyses were conducted by comparing the four genomes with a local database using blastn, and the results were analyzed using the Artemis Comparison Tool [21]. The MB11 and MB14 strains showed extensive structural similarity, while MB30 had a large inversion of approximately 1.2 Mbp compared to MB14 (Fig. 5). However, MB66 had the largest number of structural rearrangements (Fig. 5). It is worth noting that two strains with distinct infection phenotypes (MB11 and MB14) that were isolated eight years apart had largely similar genomic structures, which did not occur in the other analyzed strains.

## Conclusions

Because of the large number of infections reported for *C. pseudotuberculosis* biovar equi in recent years, sequencing and analyzing genomes for this biovar is an essential step towards new perspectives that will improve our understanding of pathogen-host interactions and facilitate the development of vaccines to eradicate the disease. The four genomes presented in this study showed structural differences, except for strains MB11 and MB14. The phylogenetic relationship is closer to other strains of the equi biovar, and other genomic characteristics, such as the GC content, number of CDSs, and tRNA and rRNA clusters, are similar to those described for other strains of the same species. Virulence factors that were previously described in the literature were identified in the analyzed genomes. In addition, *in silico* assembly of the MB11 genome was validated by an optical map of the KpnI restriction sites.

These initial data suggest that differences between types of infection should be analyzed using a reductionist approach, taking into account factors such as pathogenicity islands in each strain, the transmission method, and the entry point of the pathogen for each case, as well as expression levels and use of virulence factors specific to the bacteria, among other factors. Phylogenetic studies and the detection of small genetic changes such as SNPs and INDELs should then be performed because the bacteria have a very high gene density, and therefore, point mutations can strongly affect the biological response of the pathogen.

## Abbreviations

BHI: Brain heart infusion
CDS: Coding DNA sequence
CL: Caseous lymphadenitis
CMNR: *Corynebacterium Mycobacterium*, *Nocardia*, *Rhodococcus*
INDEL: Insertion/Deletion
Ion Torrent PGM: Ion torrent personal genome machine
PLD: Phospholipase D
SNP: Single nucleotide polymorphism
